# Feasibility and acceptability of a brief online acceptance and commitment therapy intervention to reduce fear of childbirth: A novel application of a third‐wave cognitive behavioral therapy focused on psychological flexibility and acceptance

**DOI:** 10.1111/aogs.70023

**Published:** 2025-07-30

**Authors:** Abbey A. Ashton, Jane Wilson, Pauline Slade

**Affiliations:** ^1^ Department of Primary Care and Mental Health, Institute of Population Health University of Liverpool Liverpool UK; ^2^ Liverpool Women's NHS Foundation Trust Liverpool UK

**Keywords:** acceptability, acceptance and commitment therapy, fear of childbirth, feasibility, first pregnancy, online intervention

## Abstract

**Introduction:**

Fear of childbirth (FOC) is a significant issue for many women. However, findings from studies of psychological interventions to reduce FOC are equivocal. Resource constraints highlight the need for brief interventions. Previous research demonstrated that a face‐to‐face single session acceptance and commitment therapy (ACT) intervention showed promise. The National Health Service (NHS) Long Term Plan for England highlights a wish to increase digitalization. There is a need to test online adaptations of interventions. This study aimed to assess the feasibility and acceptability, and preliminary indications of effectiveness of an online ACT group intervention for individuals who experience FOC in their first pregnancy.

**Material and Methods:**

Participants were recruited via an NHS maternity service to attend an online two‐session, small group, ACT intervention. Information on recruitment, retention, and qualitative and quantitative feedback on the sessions was gathered. Primary outcome measures (FOC and anxiety) and secondary measures (psychological flexibility and coping style) to identify potential mechanisms of change were completed pre‐intervention and 2 weeks following the second session.

**Results:**

Seven two‐session groups ran with 25 individuals participating in both sessions, indicating good feasibility. All participants were retained and provided follow‐up data. Feedback information suggested that the intervention was highly acceptable. Statistical and clinical reductions in FOC and anxiety were found. Statistically significant changes in psychological flexibility and coping style offer preliminary hypotheses regarding potential mechanisms of change.

**Conclusions:**

A two‐session online ACT group intervention tailored for FOC appears feasible and acceptable and shows promise in terms of impact. A pilot randomized controlled trial is warranted.

## Graphical abstract

Fear of childbirth (FOC) affects many mothers. There is a need for brief digital psychological interventions. This study assesses the feasibility and acceptability of a two‐session online acceptance and commitment therapy intervention for FOC. The intervention was shown to be feasible and acceptable, with reduced FOC and anxiety scores.

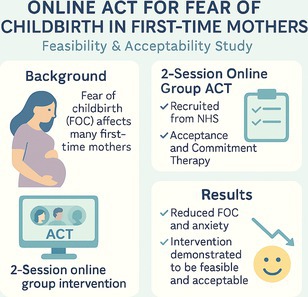

**Abbreviations**
ACT: acceptance and commitment therapy
CompACT: Comprehensive assessment of Acceptance and Commitment Therapy processes
FCQ: Fear of Childbirth Questionnaire
FOBS: Fear of Birth Scale
FOC: fear of childbirth
GAD‐7: Generalized Anxiety Disorder Scale
NHS: National Health Service
RCT: randomized controlled trial

**Key message**

A two‐session online acceptance and commitment therapy group intervention for first‐time mothers with fear of childbirth was feasible and acceptable, showing reduced fear of childbirth and anxiety, increased psychological flexibility, more adaptive coping, and reduced avoidance.

## 1 INTRODUCTION

Fear of childbirth (FOC) is thought to exist along a continuum. At the lowest level, women have no birth concerns and at the highest level, women experience extreme fear specific to giving birth, known as tokophobia.^1^ The prevalence rate of tokophobia varies across the world, with an estimated global prevalence of 14%.^2^

Severe FOC can have a negative impact on a woman's psychological well‐being during pregnancy, affect her birth experience, and lead to greater postpartum mental health difficulties.^1^ FOC is linked to increased healthcare usage, requests for medical interventions, postnatal depression, post‐traumatic stress disorder (PTSD), and low rates of breast feeding.^3^ Increased psychological distress during pregnancy can also lead to poorer developmental outcomes in children.^4^ Although parallels exist between generalized anxiety and FOC, research demonstrates that they are not synonymous and should be viewed as distinct constructs.^5^ Current guidance in England suggests that anxiety but not FOC is routinely assessed.

Research demonstrates that cognitive behavioral therapy (CBT), motivational interviewing, psychoeducation, and counseling for FOC may all reduce FOC to some extent.^6^, ^7^ However, a Cochrane review of non‐pharmacological interventions for FOC concluded that such interventions may lead to a reduction in FOC, but that the reduction may not be clinically meaningful.^8^ Therefore, currently it remains unclear which approaches are most effective and acceptable to women experiencing FOC. Additionally, the importance of collaboration between maternity and psychology disciplines to optimize support for women experiencing FOC has been highlighted.^9^ However, to the authors' knowledge, there is no current research evaluating such interdisciplinary working.

Acceptance and commitment therapy (ACT) is a transdiagnostic ‘third‐wave’ cognitive behavioral therapy.^10^ The therapeutic goal of ACT is to increase an individual's psychological flexibility, which is the ability and willingness to remain in the present moment, be accepting of unpleasant internal experiences, while choosing to behave in accordance with personal values.^10^ The opposite, psychological inflexibility, is the unwillingness, and non‐acceptance of unpleasant internal events, which lead behaviors to be dominated by attempts control the frequency or content of their thoughts or avoid these internal experiences, resulting in a loss of meaningful, value‐guided actions.^10^

A feasibility study for an eight‐session group ACT intervention for women with perinatal depression and anxiety found the intervention to be acceptable, feasible, and effective in reducing symptoms of anxiety and depression.^11^ Additionally, a randomized controlled trial (RCT) assessed the efficacy of ACT in reducing FOC and found the eight‐session intervention to be effective in reducing FOC in nulliparous women during pregnancy.^12^ It is important to consider the economic requirements to provide this level of intervention, as well as attrition rates. There is the need for shorter interventions to be offered within the NHS for FOC, as fears typically manifest toward the end of pregnancy.^13^

In our previous work, a feasibility study was conducted to assess whether a single session ACT intervention can help women manage uncertainties and FOC during first pregnancy.^14^ The intervention was found to be acceptable and feasible, and preliminary treatment signals demonstrated clinical and statistical reductions in FOC and anxiety. Although no changes in intolerance of uncertainty (IOU) were found, at baseline, high levels of IOU were uncommon in the sample. This raises an important question regarding whether IOU is a key underlying mechanism in FOC and warrants further exploration of potential mechanisms of change in FOC.

The Medical Research guidance suggests that a key source of intervention development is adapting an intervention that has been developed elsewhere to a new context.^15^ Therefore, to take our previous research forward,^14^ the current study will assess the acceptability and feasibility of an online ACT group intervention for FOC, as opposed to face‐to‐face delivery. Importantly, the NHS Long Term Plan highlights the need to increase digitalization within NHS programs.^16^

Research suggests that maternal levels of psychological flexibility play a role in the development and maintenance of perinatal mental health difficulties;^17^ however, there is limited research examining the relationship between psychological flexibility and FOC. Research demonstrates that low levels of psychological flexibility relate to utilizing avoidant coping strategies, and higher levels of psychological flexibility have been associated with the use of approach coping strategies, which are viewed as more adaptive.^18^ Therefore, the current study aims to explore changes in levels of psychological flexibility and the use of avoidant and approach coping strategies following the two‐session ACT intervention, to provide preliminary indications of change mechanisms.

### 1.1 Aims

- To assess the feasibility of a two‐session online group ACT intervention to support women experiencing FOC in first pregnancy.
- To ascertain whether the intervention is acceptable to women during their first pregnancy.
- To examine whether the outcome measures utilized are acceptable to identify preliminary treatment signals between baseline and follow‐up scores.
- To explore preliminary indications of effectiveness and identify potential mechanisms of change that may underpin any changes between baseline and follow‐up.

## 2 MATERIAL AND METHODS

### 2.1 Design

This feasibility study used a pre‐post mixed methods design and was informed by the Medical Research Council's guidance for the development and evaluation of complex interventions.^15^ Qualitative feedback regarding participant experiences for session one and two was gathered, and standardized outcome measures were administered, gathering quantitative data at two time points: T1 (pre‐session) and T2 (two weeks following session two).

### 2.2 Participants

Based on previous feasibility studies which evaluated interventions for FOC,^14^ the current study aimed to recruit a maximum of 50 women, with an estimated engagement rate of 50%, resulting in a final sample of 25 women.

The inclusion criteria were as follows: women had to be in their first pregnancy, carrying a single pregnancy, receiving care from Liverpool Women's Hospital, and self‐reporting worries in relation to childbirth. Participants were required to have a good understanding of English, be over the age of 18 years, and be between 11 and 34 weeks pregnant at the time of recruitment.

The exclusion criteria were individuals aged under 18 years or who were carrying multiple pregnancies, who were under the care of the hospital's fetal medicine unit. Individuals were also excluded if they were in receipt of care from psychiatric services for severe and enduring mental health difficulties.

### 2.3 Recruitment

Community midwives were provided with an information card detailing the inclusion/exclusion criteria and a QR code to share with eligible individuals who expressed FOC during antenatal appointments. The QR code directed women to an online survey which displayed the recruitment poster and an expression of interest form and allowed women to provide consent to be contacted by the researcher. The recruitment poster and the expression of interest form were also posted on the hospital's social media pages. Women could self‐refer in response to the advertisement by completing the online expression of interest form. The eligibility of self‐referring individuals was checked with the Consultant Midwife (JW).

Upon completion of the expression of interest form, the researcher emailed women, checked eligibility, and provided information about the research. Women who wished to take part were sent a participant information sheet, an online consent form, and pre‐session measures with completion needed before a first session. Women were also sent instructions for accessing the Zoom platform and were informed that the Zoom meeting links would be sent out via email on the morning of the session date.

### 2.4 Materials

The ACT session materials used in our previous study were adapted for an online session delivery.^14^ The session materials included two “Coping with Worries and Fears of childbirth” presentations and a self‐help toolkit. To equip the researcher with the necessary skills and knowledge to deliver the sessions and adapt the materials, in addition to their Doctorate in Clinical Psychology, first author (AA) completed an ACT training course. The materials were developed in partnership with two experts by experience, recruited through the Hospital's Maternity Voices Partnership and in consultation with a Consultant Clinical Psychologist (PS) and midwifery supervisor (JW).

The presentations used incorporated didactic learning methods via ACT informed psychoeducation and experiential exercises. The presentations mapped onto the “be present, be open and do what matters” approach,^10^ and were tailored to coping with fears regarding childbirth. Additional information regarding the ACT skills and concepts drawn upon in the two sessions are detailed in Supplementary Material (Table S1).

### 2.5 Procedure

All group sessions were facilitated by the first author (AA) and the second author (JW). The sessions ran on weekday evenings via the Zoom video‐conferencing platform, with the first group session lasting 2 h and the second group session lasting 1 h.

During both sessions, time was provided for group discussions, reflections, and questions. The Consultant Midwife offered specialist knowledge in relation to any specific birth‐related queries. Following each session, women completed the ACT‐session evaluation form anonymously. The self‐help toolkit offering additional ACT coping strategies and links to ACT resources was introduced in session one and was sent by email. The second session ran two weeks after the first session. Two weeks after attending the second session, women were requested to complete follow‐up measures. In line with ethical approval, women who completed the follow‐up measures were provided with a £10 Amazon gift voucher as reimbursement for their time.

### 2.6 Measures

#### 2.6.1 Acceptability

To ascertain the acceptability of the intervention, an anonymous online feedback form was completed by participants after both sessions. This covered feedback on clarity, organization, interest, and usefulness of the intervention, as well as information regarding use and experience of the self‐help toolkit. It also requested suggestions for improvement and any further comments.

#### 2.6.2 Demographic information

Demographic data were obtained from participants in relation to their age, marital status, ethnicity, education level, and pre‐pregnancy employment status.

#### 2.6.3 Primary outcome measures

FOC was assessed using two measures and anxiety symptoms were assessed using one measure. These measures were administered at T1 and T2.

##### Fear of childbirth (FOC)

###### Fear of Birth Scale (FOBS)^19^

The FOBS consists of two visual analogue scales, each with a 0–100 range, which are summed and averaged to get a score. Participants rate degree of worry (calm/worried) and fear (no fear/strong fear) about the approaching birth with a single question: “How do you feel right now about the approaching birth?” The cut‐off to identify fear of childbirth is a score of 50. The FOBS has high internal consistency and is a valid measure for measuring FOC with sensitivity of 89% and specificity of 79%.^19^

###### Fear of Childbirth Questionnaire (FCQ)^20^

The FCQ is a 20‐item scale designed to measure FOC in English‐speaking women. Items are rated on a 4‐point scale. Total scores range from 0 to 60, with higher scores indicating a higher level of fear. There is evidence of good construct validity and internal reliability information for UK populations.^20^

##### Anxiety symptoms: Generalized Anxiety Disorder Scale (GAD‐7)^21^

The GAD‐7 is a seven‐item scale that measures anxiety levels. The scale ranges from 0 = not at all to 3 = nearly every day. Total scores range from ‘0–21’. A score of >10 represents clinical levels of anxiety. Sensitivity is 89% and specificity 82%.^21^

#### 2.6.4 Secondary outcome measures

The secondary outcome measures were used to examine indications of potential mechanisms of change that may underpin any changes in T1 and T2 scores on primary outcome measures. A measure of psychological flexibility and a measure of coping style were administered at T1 and T2:

##### Psychological flexibility: The Comprehensive assessment of Acceptance and Commitment Therapy processes (CompACT)^22^

The CompACT is a 23‐item scale designed to measure psychological flexibility. This is the ability to be fully aware and accepting of all internal experiences, such as thoughts, feelings, and act in a manner that is consistent with our personal values.^10^ Items are rated on a 7‐point scale. Total scores range from 0 to 138. This measure has demonstrated convergent, discriminant, and concurrent validity.^22^

##### Brief—Coping Orientation to Problems Experienced Inventory (Brief COPE)^23^

The Brief‐COPE is a 28‐item questionnaire measuring effective and ineffective ways of coping with a stressful life event. The scale can determine primary coping styles as either approach or avoidant coping.^24^ Total scores on both avoidant and approach coping subscales range from 0 to 48. This scale is well‐validated for use in the general population and has been shown to have acceptable reliability during pregnancy.^23^

### 2.7 Data analysis

Descriptive statistics were used to collate demographic data. Measures of skewness, kurtosis, and analysis of histograms allowed for the normality of the data to be assessed. Scores on the FOBS, CompACT and Brief‐COPE at T1 and T2 were compared using paired t‐test due to the normality of the data. Scores of the FCQ and the GAD‐7 at T1 and T2 were compared using the Wilcoxon Matched‐Paired Signed‐Rank Test due to the non‐normality of the data. The threshold for statistical significance was *p* < 0.05. Hedges g statistic was used to inform effect size. Effect sizes were classified as follows, *g* ≤ 0.5 is a small effect size, 0.5 < *g* < 0.8 is a medium effect size and 0.8 ≤ *g* < 1.3 is a large effect size. Analysis of reliable change and clinically significant improvement were calculated using Reliable Change Index methodology,^25^ alongside the “How to do it V3.1 with graph.xlsm”.^26^ The ACT session evaluation form data was described using descriptive statistics and qualitative feedback was analyzed using content analysis.^27^

## 3 RESULTS

### 3.1 Recruitment and attendance

As displayed in Table 1, 46 women expressed an interest in attending the sessions, 26 women attended session one, and 25 of these women also attended session two. All 25 women who attended both sessions completed T1 and T2 measures and completed the feedback questionnaires. One woman completed T1 measures and attended session one; however, she was unable to attend session two as she had given birth by this date. Over seven months, seven two‐session groups ran. The range of women attending each session ranged from one to seven, with an average of four per session.

**TABLE 1:** Recruitment data.

Source	Expression of Interest *n* (%)	Proportion of women who attended session one	Proportion of women who attended session two	Proportion of women who completed T1 measures	Proportion of women who completed T2 measures
Midwifery referral	10 (21.7)	7/10	7/7	7/10	7/7
Self‐referral: Clinic poster	6 (13.1)	2/6	2/2	2/6	2/2
Self‐referral: Social media	30 (65.2)	17/30	16/17	17/30	16/17
Total	46 (100)	26/46	25/46	26/46	25/46

### 3.2 Participants

Participant demographic data are described in Table 2. The age of women in the sample were comparable to national maternity data.^28^ However, differing from national comparative data,^29^, ^30^, ^31^ more women in the sample identified as being White British, were married, had a higher level of education, and had higher rates of employment.

**TABLE 2:** Participant characteristics (*n* = 26).

	Mean (*SD*)	Range
Age (years)	31.2 (2.63)	27–38
Gestation at T1 (weeks)	26.9 (5.84)	18–34
Gestation at T2 (weeks)	31.3 (5.62)	22–39

	Total, *n* (%)
Marial status	
Married	17 (65.4)
Cohabiting	9 (34.6)
Education level	
Education (graduate/postgraduate)	20 (76.9)
Education (A‐levels, vocational)	4 (15.4)
Education level (GCSEs)	2 (7.7)
Pre‐pregnancy employment status	
Paid, full‐time	25 (96.2)
Unemployed	1 (3.8)
Other (paid, part‐time/student/voluntary work/self‐employed)	0
Ethnic origin	
White British	23 (88.5)
White other	1 (3.8)
Mixed other	1 (3.8)
Other	1 (3.8)
Black/African/Caribbean	0
Asian (Indian, Bangladeshi, Pakistani, … Chinese, any other Asian background)	0

### 3.3 Descriptive statistics

As attendance at session two was almost complete, with the loss of only one person, the descriptive data for the 25 women who completed session one and two is provided in Table 3. Regarding clinical cut‐off scores on the FOBS, at T1, 20 of 26 women reported clinical levels of FOC and seven reported “moderate” or above levels of anxiety. At T2, five of 25 women reported clinical levels of FOC and two reported moderate levels of anxiety at T2.

**TABLE 3:** T1 and T2 descriptive statistics.

Variable	Pre‐session scores—T1 (*n* = 25)		Post‐session scores—T2 (*n* = 25)	
	Mean (SD)	Median (range)	Mean (SD)	Median (range)
Fear of Birth Scale (0–100) (FOBS)^a^				
Q1. Calm–worried	60.64 (20.45)		36.84 (16.55)	
Q2. No fear–fear	57.00 (21.58)		35.92 (18.43)	
Average	58.8 (19.5)	65 (86.5)	36.3 (17.04)	40 (73.5)
Fear of Childbirth Questionnaire (FCQ)^a^				
Total score	33.12 (7.88)	32 (5.50)	25.48 (10.82)	26 (15.00)
Anxiety Symptoms (GAD‐7)^a^				
Total score	5.8 (5.10)	4 (16)	3.7 (3.49)	3 (12)
Comprehensive Assessment of ACT Processes (CompACT)^b^				
Valued action	34.88 (3.83)		38.24 (6.01)	
Openness to experiences	27.16 (8.13)		38.68 (9.52)	
Behavioral awareness	16.12 (6.33)		20.04 (7.08)	
Total score	78.16 (13.90)		96.96 (17.90)	
Brief Coping Orientation to Problem Experiences (COPE)				
Approach Coping^b^	32.12 (7.30)		37.08 (6.31)	
Avoidant Coping^a^	20.96 (5.06)		18.92 (3.89)	

Abbreviation: GAD‐7, Generalized Anxiety Disorder‐7.

^a^ Reduction in scores indicates improvement.

^b^ Increase in scores indicate improvement.

### 3.4 Paired *t*‐test/Wilcoxon matched‐paired signed‐rank test

#### 3.4.1 Primary mental health outcomes

In terms of FOC measures, the average score on the FOBS was significantly lower at T2 (*M* = 36.3) than at T1 (*M* = 58.8, *T* = 6.91, *p* < 0.001); a large effect size was found (g = 1.34). On the FCQ, women's average scores were significantly lower at T2 (*Mdn* = 26) than at T1 (*Mdn* = 32, *Z* = −3.45, *p* = 0.001) and a medium effect size was found (*g* = 0.65). On average, women's anxiety scores were significantly reduced from T1 (*Mdn* = 4) to T2 (*Mdn* = 3, *Z* = −2.53, *p* = 0.012). The effect size was medium with a Hedges' g of 0.51. See Table 4.

**TABLE 4:** Within‐group analysis (*n* = 25).

Measure	*Z*‐score	*T*‐score	*p*‐value	Effect size (g)	95% Confidence interval
FOBS	–	6.91	<0.001	1.34	15.74, 29.13
FCQ^a^	−3.45		0.001	0.65	−12.0, −4.0
GAD‐7^a^	−2.53		0.012	0.51	0.56, 3.67
CompACT total	–	−6.31	<0.001	−1.22	−24.94, −12.65
Valued action		−3.11	0.005	−0.60	−5.58, −1.13
Open to experiences		−6.89	<0.001	−1.33	−14.96, −8.07
Behavioral awareness		−3.95	<0.001	−0.76	−5.95, −1.87
COPE					
Approach coping	–	−4.91	<0.001	0.95	−2.0, 2.0
Avoidant coping	–	2.07	0.049	0.40	−2.0, 0.5

*Note*: *p* ≤ 0.05.

Abbreviations: CompACT, Comprehensive Assessment of Acceptance and Commitment Therapy Processes; FCQ, Fear of Childbirth Questionnaire; FOBS, Fear of Birth Scale; GAD‐7, Generalized Anxiety Disorder‐7.

^a^ Non‐parametric test; medians analyzed.

#### 3.4.2 Secondary outcome measures

Women's overall psychological flexibility scores significantly improved following the intervention (T2: *M* = 96.96) (T1: *M* = 86.16, *T* = −6.31, *p* < 0.001) and a large effect size was found (*g* = −1.22). Use of approach coping strategies had significantly increased following the intervention (T2: *M* = 37.08) (T1; *M =* 32.12, *T* = −4.91, *p < 00.1*) and effect size was found to be large (*g* = 0.95). A significant decrease in use of avoidant coping strategies was found at T2 (*M* = 18.92) in comparison at T1 (*M* = 20.96, *T* = 2.07, *p* = 0.049) with a moderate effect size (*g* = 0.40). See Table 4.

### 3.5 Future trial sample size calculation

G*Power v3.1 was used to estimate the sample size needed to detect statistically significant differences in mean FOBS scores at baseline and follow‐up using a RCT design. It was estimated that nine participants would be required in each of the experimental and control groups, due to the large within‐group effect size on the FOBS (α level *p* = 0.05, power 90%).

### 3.6 Reliable change and clinically significant change

The reliable change index and clinically significant change analysis shows a reliable improvement and clinically significant reduction in FOC scores for 18 of the 25 women (72%) and in anxiety symptoms for four women (16%). Importantly, none showed deterioration. This analysis demonstrates that the intervention led to greater clinically significant changes specific to FOC, as opposed to more general anxiety symptoms (Table 5).

**TABLE 5:** Reliable change index (RCI) and clinically significant change (CSC) for T1 and T2 primary mental health outcome measures (*n* = 25).

Variable	T1 mean (SD)	T2 mean (SD)	Reliability co‐efficient (a)	Normative mean (SD)	RCI	No change, *n* (%)	Improved, *n* (%)	Deteriorated, *n* (%)	CSC, *n* (%)
FOBS	58.8 (19.5)	36.3 (17.04)	0.91	41.00 (21.00)	16.27	7 (28%)	18 (72%)	0	18 (72%)
GAD‐7	5.84 (5.10)	3.70 (3.49)	0.89	3.20 (3.50)	4.69	21 (84%)	4 (16%)	0	4 (16%)

Abbreviations: FCQ, Fear of Childbirth Questionnaire; FOBS, Fear of Birth Scale; GAD‐7, Generalized Anxiety Disorder‐7.

### 3.7 ACT session feedback

The feedback provided by the women was very positive, with most women rating both sessions as *extremely* or *very useful* (session one: 88%, session two: 96%) and *interesting* (session one: 92%, session two: 96%). Most women rated the clarity of explanations provided throughout both sessions as *extremely clear* (session one: 80%, session two: 84%) and indicated that they were *very confident* (session one: 52%, session two: 60%) in their ability to apply the strategies in their daily lives (Table 6).

**TABLE 6:** Session one and two feedback: clarity, organization, interest, usefulness, and understanding.

Question and response options	Session one	Session two
	Frequency (%)	Frequency (%)
How clear were the session objectives?		
Extremely unclear	0	0
Somewhat unclear	0	0
Neither clear nor unclear	0	0
Somewhat clear	5 (20%)	4 (16%)
Extremely clear	20 (80%)	21 (84%)
How well organized was the session?		
Not well at all	0	0
Slightly well	0	0
Moderately well	1 (4%)	0
Very well	7 (28%)	6 (24%)
Extremely well	17 (68%)	19 (76%)
How interesting was the session?		
Not interesting at all	0	0
Slightly interesting	0	0
Moderately interesting	2 (8%)	1 (4%)
Very interesting	12 (48%)	10 (40%)
Extremely interesting	11 (44%)	14 (56%)
How useful was the session?		
Not at all useful	0	0
Slightly useful	0	0
Moderately useful	3 (12%)	1 (4%)
Very useful	9 (36%)	10 (40%)
Extremely useful	13 (52%)	14 (56%)
How confident are you that you will be able to apply some of the strategies discussed today into your daily life?		
Not at all confident	0	0
Somewhat confident	2 (8%)	0
Moderately confident	6 (24%)	2 (8%)
Very confident	13 (52%)	15 (60%)
Extremely confident	4 (16%)	8 (32%)

	Frequency (%)
How clearly was “be present” explained?	
Extremely unclear	0
Somewhat unclear	0
Neither clear nor unclear	0
Somewhat clear	3 (12%)
Extremely clear	22 (88%)
How clearly was “be open” explained?	
Extremely unclear	0
Somewhat unclear	0
Neither clear nor unclear	0
Somewhat clear	3 (12%)
Extremely clear	22 (88%)
How clearly was “do what matters” explained?	
Extremely unclear	0
Somewhat unclear	0
Neither clear nor unclear	0
Somewhat clear	3 (12%)
Extremely clear	22 (88%)
Which strategies appealed the most?	
Mindfulness and being present	18 (72%)
Observing self and being open	12 (48%)
Value driven behaviors	11 (44%)

Most women had referred to the self‐help toolkit during the two‐week gap once or twice (92%). Approximately half of those who referred to the toolkit did so 2–3 times (52%) and the remaining women referred to it once during the 2 weeks. Most women (78%) reported that they felt *very* or *extremely confident* in their ability to make use of the toolkit.

Content analysis of the 25 comments provided across session one and two feedback forms generated 12 themes. The feedback provided was positive, with women expressing gratitude for the sessions and reporting the sessions to be informative and enjoyable, and the ACT techniques being helpful for coping with birth‐related anxieties, as well as more general experiences of anxiety. Detail is available in Supplementary Material (Table S2).

## 4 DISCUSSION

This study used a mixed methods design to explore the feasibility and acceptability of a two‐session online ACT intervention to support the mental well‐being of women experiencing FOC during their first pregnancy.

The current study found it possible to recruit women experiencing fears regarding childbirth via self‐referral and signposting from community midwives. Over the 7‐month recruitment period, 46 women expressed an interest in taking part, 26 women were eligible and signed up to participate. Of the 26 women who signed up, 25 women took part in both sessions and completed follow‐up questionnaires. One woman did not complete session two as she gave birth earlier than expected. The very high retention rates support the feasibility of recruiting and retaining women in their first pregnancy who experience FOC to a two‐session online ACT‐informed group intervention. The retention rates of the two‐session online group intervention are typically higher than those reported within longer psychological interventions delivered within the perinatal period.^12^, ^32^, ^33^

Although the results are preliminary, the almost complete retention rate, including return of research measures in the current study, is promising. In summary, a brief two‐session online group ACT intervention to support women in first pregnancy with FOC appears feasible.

Analysis of the post‐session feedback forms indicated evidence for the acceptability of the intervention. Firstly, the feedback forms were completed following session one and two by all women. This suggests that quantitative and qualitative evaluative feedback is acceptable. Women's experience of session one and two was similarly positive, with all women reporting that the sessions were useful and interesting and found the different strategies appealing, and most women reported the ACT strategies were explained clearly, and that they had confidence in their ability to apply the ACT strategies into their daily life.

First, significant reductions in FOC and anxiety following the intervention were found. The post intervention effect size on the FOBS was large and on the FCQ was medium. The effect size on the FOBS was slightly larger than that reported for the face‐to‐face single session ACT intervention for FOC,^14^ and higher than that reported for internet‐based CBT interventions for FOC, where the effect size was medium.^33^ Over two thirds of women demonstrated reliable and clinically significant changes in relation to their FOC. This is important as while a recent review of FOC interventions indicated positive changes, these were questionable in terms of clinically meaningful change.^8^

In relation to changes in general anxiety symptoms, measured by the GAD‐7, the post intervention effect size was medium. As the intervention targeted women experiencing FOC, rather than generalized anxiety, and as these concepts are not synonymous,^5^ it is not surprising that only 18% presented with moderate levels of anxiety symptoms. Of the women who experienced a reduction in anxiety symptoms following the intervention, 16% were found to have reliable and clinically significant reductions and no women experienced an increase in general anxiety symptoms following the intervention. The specificity of the change in relation to FOC rather than general anxiety suggests the importance of interventions to target this aspect and reinforces evidence in the literature that these are distinct dimensions of distress requiring specific identification and intervention.^5^

A significant increase in psychological flexibility following the intervention was found, with a large within‐group effect size. This demonstrates the validity of the intervention, given that this is the key process targeted in ACT.^10^ This finding is in accordance with the ACT theoretical model, which suggests that increases in levels of psychological flexibility are associated with reductions in psychological distress.^10^ The within‐group psychological flexibility effect sizes are comparable to those reported for face‐to‐face 8‐session ACT group intervention for perinatal mood and anxiety difficulties.^12^ Finally, significant increases in the use of approach coping strategies and significant reductions in the use of avoidant coping strategies were found following the intervention. The effect size for approach coping was large, and for avoidant coping was medium.

These findings provide preliminary indications of mechanisms of change. Research has found that higher levels of psychological flexibility led to an increase in approach coping, a reduction in avoidant coping, and a reduction in psychological distress.^34^ Therefore, it is possible that in the current study, increases in psychological flexibility led to an increase in approach coping and a reduction in avoidance via increases in willingness and acceptance of thoughts and feelings, which in turn led to a reduction in fear. Although these results are preliminary, they generate important theoretical hypotheses in relation to psychological flexibility and its potential role in facilitating adaptive coping strategies as the mechanism of change in FOC following the ACT intervention.

The preliminary treatment signals suggest that the two‐session online ACT group intervention may be beneficial for women experiencing FOC during first pregnancy. The National Institute of Health and Care Research framework for developing and evaluating complex interventions suggests that the next stage would be to carry out an external pilot RCT to test the feasibility of recruitment and randomization within this population.^15^ The power calculation completed in the current study can be used to ensure future RCTs are adequately powered.

A strength was the integrated working between psychology and midwifery, as the intervention sessions were jointly run. It has been suggested that interventions delivered to support perinatal mental health can be improved by support offered via midwives.^35^

The lack of a control group and small sample size limits the generation of concrete conclusions about intervention efficacy. As the follow‐up period was limited to two weeks, longer‐term effects of the intervention are unknown. Additionally, it is important to reflect on the possible response bias via demand characteristics. As the T1 and T2 measures were emailed to the women by the first author/group facilitator (AA), women may have altered their responses to fit their assumptions of the research hypotheses or to please the facilitator/researcher. Finally, the diversity of the sample was limited, with an overrepresentation of white women, who were married with high educational attainment and in full‐time employment.

Within England, the NHS Long Term Plan emphasizes the need for increasing specialist mental health support for women and families during the perinatal period.^16^ FOC is a major clinical concern as the distress associated with FOC has extensive implications for women, children, and healthcare services.^1^ Research assessing psychological interventions to reduce FOC has been deemed a priority in an expert consensus statement.^36^ Within the UK, Maternal Mental Health Services have recently been developed that offer specialist interventions for women experiencing FOC. However, it has been demonstrated that FOC interventions that have been researched to date may not lead to a clinically meaningful reduction in FOC.^8^ Therefore, the feasibility and acceptability of a two‐session online ACT group intervention that has been demonstrated, along with preliminary indications of clinically significant improvements in FOC and potential mechanisms of change, is timely and important.

As part of an external pilot with a randomized controlled design, it would be of value to include an economic evaluation. Due to the brevity of the intervention, it is highly likely to be cost‐effective. Future research may also wish to utilize a longitudinal research design to explore women's application of ACT skills during labor and aim to recruit a more diverse sample to improve the generalizability of the findings.

## 5 CONCLUSION

The feasibility and acceptability of a two‐session online group ACT intervention to support women who experience FOC in their first pregnancy has been demonstrated. Positive preliminary treatment signals have also been demonstrated, with decreases in FOC and anxiety symptoms. Potential mechanisms of change have been identified, with increases in psychological flexibility levels and use of more adaptive coping strategies and reductions in the use of less adaptive avoidant coping strategies. Future research needs to move toward RCT designs, and the intervention must be provided to a more diverse sample of first‐time pregnant individuals to establish general efficacy.

## AUTHOR CONTRIBUTIONS

Pauline Slade had the original idea for this study. Abbey Ashton and Pauline Slade were responsible for the design of this study. Abbey Ashton and Jane Wilson co‐delivered the intervention. Jane Wilson aided in the recruitment of participants into the study. Abbey Ashton conducted data collection, extraction, and performed the analyses. Pauline Slade and Jane Wilson offered supervision to Abbey Ashton throughout the recruitment and intervention delivery period. All authors contributed to the interpretation of the data. Abbey Ashton drafted the initial manuscript, which was reviewed by Pauline Slade, who also contributed to writing the manuscript. Abbey Ashton revised the manuscript with supervision from Pauline Slade. All authors have approved the submitted version of the manuscript.

## CONFLICT OF INTEREST STATEMENT

We declare that we have no financial or other interest in any commercial product mentioned in our review. There are no conflicts of interest related to consultancies, stock ownership, or patent‐licensing arrangements.

## ETHICS STATEMENT

University of Liverpool Sponsorship (UoL001752), HRA and Health and Care Research Wales approval (IRAS: 323825; REC Reference: 23/PR/0083) were granted prior to data collection on February 16, 2023.

## Supporting information


Table S1.

Table S2.

## Data Availability

The data that support the findings of this study are available on request from the corresponding author. The data are not publicly available due to privacy or ethical restrictions.
