# Supplementary material for: Feasibility and acceptability of a brief online acceptance and commitment therapy intervention to reduce fear of childbirth: A novel application of a third‐wave cognitive behavioral therapy focused on psychological flexibility and acceptance
Source: Acta Obstet Gynecol Scand. 2025 Jul 30;104(10):1939–48. doi: 10.1111/aogs.70023 (PMC12451198; doi:10.1111/aogs.70023)
Supplement: Supplementary file 1 — Table S1. [file AOGS-104-1939-s001.docx]

| **Table S1 – SUPPLEMENTARY MATERIAL**  **Description of ACT skills incorporated into the sessions** | | |
| --- | --- | --- |
| **Session Number** | **ACT concept/skill** | **Description** |
| One | Experiential avoidance and the happiness trap | The ACT underlying theoretical model was presented and the concept of experiential avoidance and the happiness trap was described. The pink elephant paradox and the beach ball metaphor was used to aid understanding. |
| One and two | Present moment awareness | The basic components of mindfulness were introduced and practiced using the mindfulness to thoughts exercise “leaves on the stream” in session one.  This skill was recapped in session two and practised using a colour breathing exercise. Time was given for problem solving, questions or reflections regarding this skill. |
| One and two | Self-as-context | The self-as-context concept and skill was introduced in session one. The “stage-show” metaphor was used to aid understanding.  This was recapped in session two. The “sky and weather” metaphor was introduced in session two to aid understanding. Time was given for problem solving, questions or reflections regarding this skill. |
| One and two | Defusion | Defusion vs fusion concept was introduced in session one. Two metaphors were drawn upon to aid understanding, these included: the “master storyteller”, and the “thought bully”. Two experiential exercises were also used to practise defusion: the “pushing away paper” exercise and the “I’m having a thought that…” exercise.  This was recapped in session two. Time was given for problem solving, questions or reflections regarding this skill. |
| One and two | Acceptance | The concept of acceptance vs avoidance was introduced in session one. The “tug of war with a monster” and “struggle switch” metaphors were used to aid understanding.  This was recapped in session two. Willingness vs wanting was also introduced. The cactus metaphor was using to explain the value of accepting difficult thoughts and feelings. Time was given for problem solving, questions or reflections regarding this skill. |
| One and two | Values | Within session one, the concept of personal values was introduced and explained. Participants were also invited to reflect on their personal values. The sweet spot exercise was used to elicit participants' personal values. A discussion on parenthood-related values was also facilitated. |
| One and two | Committed action | Committed action was introduced towards the end of session one. Participants were encouraged to engage with committed action between session one and two. They were asked the question “What is a small, simple, easy thing you could do in the next 24 hours that will take you a little further towards your values of motherhood?”  In the second session, women were invited to discuss their experience of engaging in committed action. Additionally, the concept of workability and the role of workability in relation to committed action was introduced. Participants engaged in an exercise whereby they were required to reflect on the workability of various case studies. |
| Two | Self-compassion | At the end of session two, participants were invited to engage in a self-compassion exercise, named “kind hands”. This exercise encourages participants to acknowledge and accept their difficult emotions and hold themselves and their bump kindly. |
