# Supplementary material for: Feasibility and acceptability of a brief online acceptance and commitment therapy intervention to reduce fear of childbirth: A novel application of a third‐wave cognitive behavioral therapy focused on psychological flexibility and acceptance
Source: Acta Obstet Gynecol Scand. 2025 Jul 30;104(10):1939–48. doi: 10.1111/aogs.70023 (PMC12451198; doi:10.1111/aogs.70023)
Supplement: Supplementary file 2 — Table S2. [file AOGS-104-1939-s002.docx]

| General comments | | |
| --- | --- | --- |
| Content (code) | Quotes | Frequency |
| Helpful | *“I felt I have benefitted from them”. “Very helpful, thank you!”* | 10 |
| Gratitude | *“Very powerful session, makes you stop and think, thank you”. “I appreciate the time and information given to me”* | 9 |
| Learning of new skills | “*It was really useful to reframe my thinking and gave me some techniques to practice”. “I think these new skills were useful and I can see them benefitting me during pregnancy”* | 6 |
| Informative | “*Very informative, made me think of things that I wouldn't have otherwise thought of” “I found the methods discussed really informative”* | 5 |
| Enjoyment | *“I enjoyed the sessions a lot” I really enjoyed the chance to speak to other women in the same position and hear their stories.* | 5 |
| Generalisability | *“..I feel they’ll not only be useful for anxieties around childbirth but in general..” “I have found the sessions very helpful not only for worries about birth, but coping with more general worries as well.”* | 4 |
| Accessibility | *“Felt it was laid out great” “I think it was extremely well presented”* | 3 |
| Participation | *“X gave us chance to openly speak” “X gave us chance to speak freely”* | 2 |
| Anonymity | *“The padlets were great for jotting down ideas anonymously”. “I liked the interactiveness of the whiteboard, which meant we were getting involved but not necessarily having to speak or share own thoughts, it was still quite confidential.”* | 2 |
| Enthusiasm ^[[1]](#footnote-1)^ | “*I'm looking forward to the next session”* | 2 |
| Supportive | *“X was really supportive, and I felt listened to”* | 1 |
| Increasing availability | *“I think all women who are pregnant should be offered these sessions.”* | 1 |
| Integration of psychology and midwifery | “*Really liked how x the midwife was involved too alongside x”* | 1 |

1. Enthusiasm code is generated from session one feedback only. [↑](#footnote-ref-1)
